# A Case of an Uncommon Lower Lip Swelling

**DOI:** 10.7759/cureus.48901

**Published:** 2023-11-16

**Authors:** Ahmad Firdaus Habib Rahman, Khoo S Jiun, Kanivannen Arasu, Khairunnisa M Zuhaidi, Avatar Singh Mohan Singh

**Affiliations:** 1 Otorhinolaryngology-Head & Neck Surgery, Hospital Taiping, Taiping, MYS; 2 Pathology, Hospital Taiping, Taiping, MYS

**Keywords:** neurilemmoma, lower lip swelling, schwann cell, head and neck tumors and diseases, nerve schwannoma

## Abstract

A woman in her fifty-seventh year appeared with a painless swelling on her left lower lip that had been present for three years. The size of the swelling was noticeably increasing in size over time. On examination, there was a lump that was firm, non-tender, and measured 2 cm x 1 cm above the lower lip. The oral cavity, as well as the intraoral mucosa, were normal and unremarkable in appearance. As fine-needle aspiration cytology revealed a spindle cell lesion, she had an excision biopsy performed, followed by bilateral advancement flap closure. Histopathological examination (HPE) revealed a schwannoma in the tissue sample. Schwannomas are rare, benign neural tumors originating from Schwann cells. They are mainly asymptomatic and have a moderate rate of growth. Excision is the recommended treatment for schwannomas. Proper surgical planning and postoperative monitoring are crucial for optimal wound healing and complete recovery.

## Introduction

Peripheral nerve tumors are mostly benign and consist of neurofibroma and schwannoma (neurilemmoma) [1], both of which originate from Schwann cells [1]. Schwannoma of the lower lip is very rare. There is no documentation of such a case in Malaysia as of now. The tongue has the highest incidence at the intraoral location, followed by the palate, buccal mucosa, lip, and gingiva [2]. Herein, we would like to report a case of a 57-year-old who presented with lower lip swelling, which turned out to be a case of Schwannoma. It usually presents as a painless, slow-growing tumor. Given its rarity, it is seldom included in the list of differentials of such swelling. Thus, a diagnosis is often made after its histological examination.

## Case presentation

**Figure 1:**
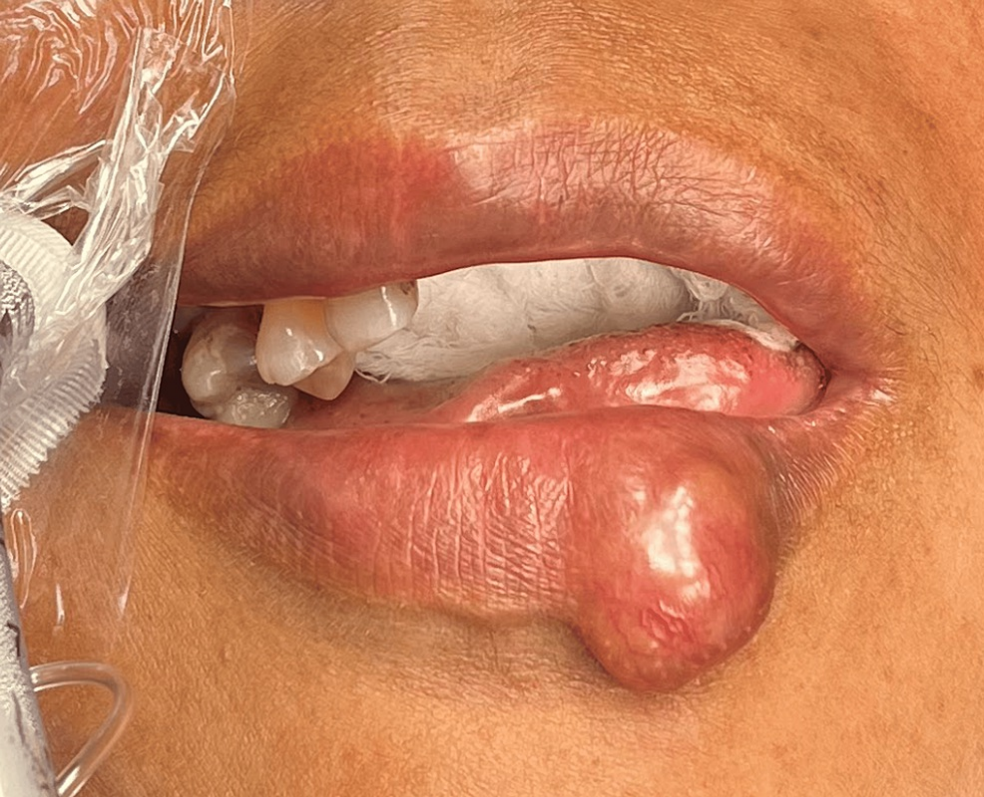
Lower lip swelling

**Figure 2:**
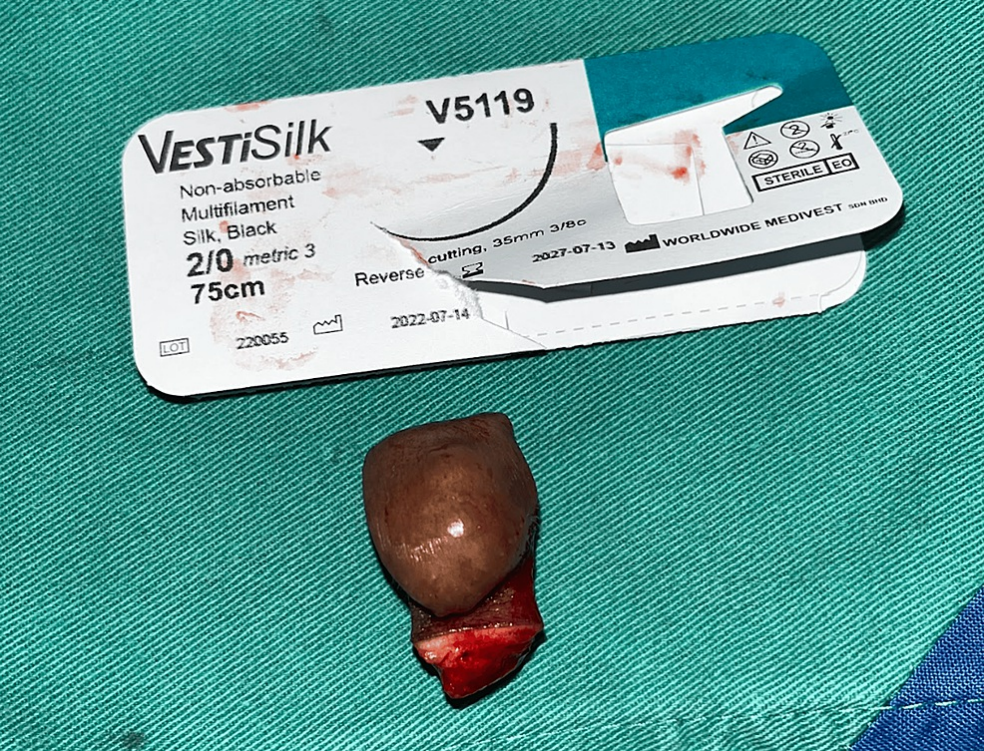
Swelling removed (VestiSilk for size comparison)

This patient was a 57-year-old woman with underlying hypertension and dyslipidemia. She presented with a painless left lower lip swelling for three years. The swelling was gradually increasing in size. There was no history of insect bite, lip biting, or trauma. There was never an ulcer or discharge as well. On examination, there was a firm swelling measuring 2 cm x 1 cm over the inferior lip (Figure 1 and Figure 2)*.*

**Figure 3:**
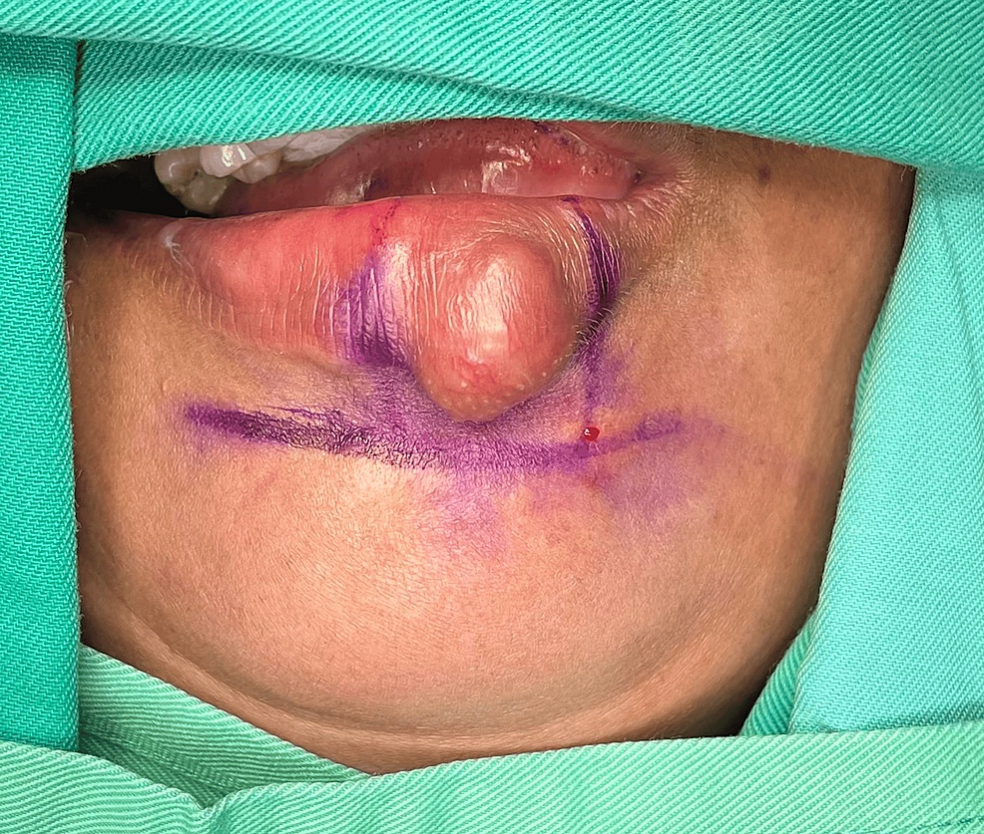
The cutaneous skin on both the lateral and inferior aspects of the unaffected portion of the lower lip swelling are delineated with a linear marking with the horizontal incision marked over the mental crease

**Figure 4:**
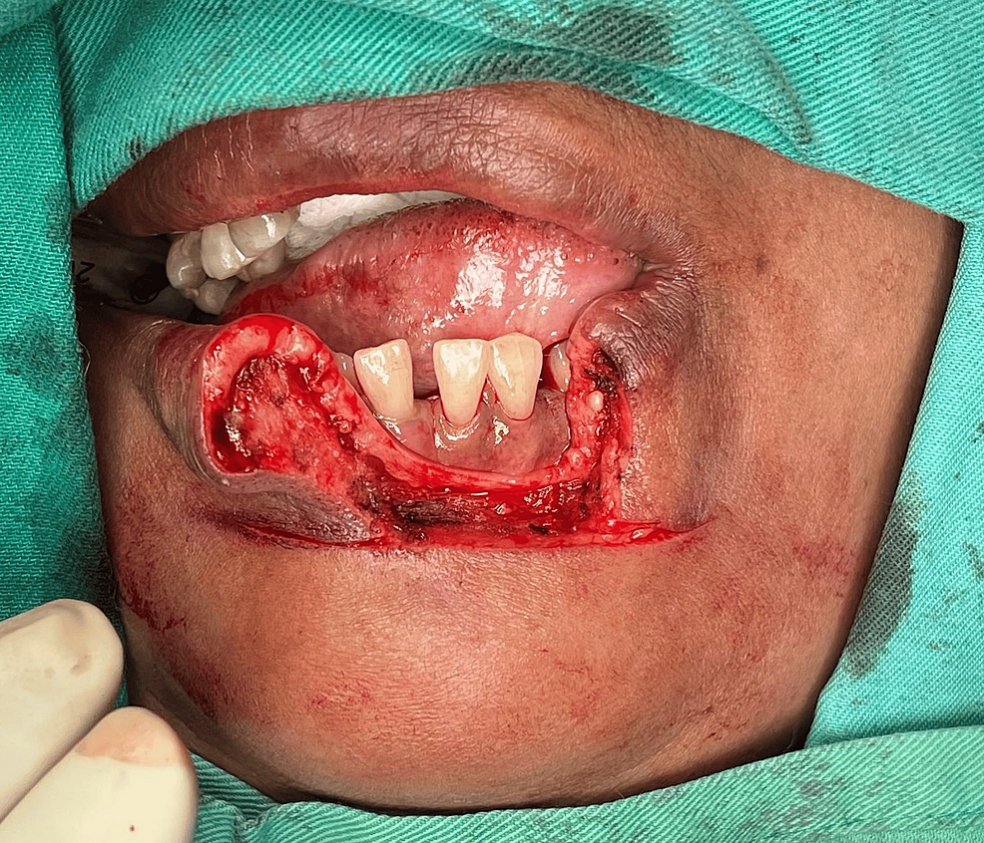
An incision was performed at the cutaneous skin marking followed by the mucosa of the oral cavity, and the lesion was excised

**Figure 5:**
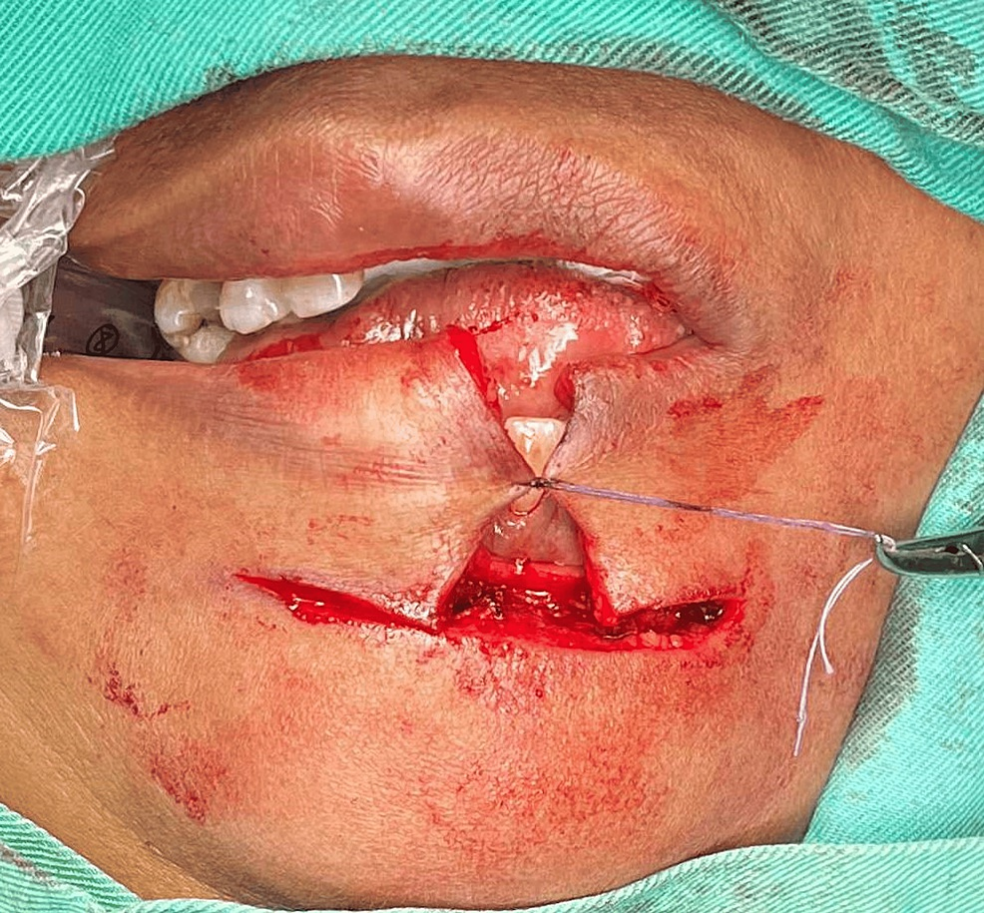
The lip defect was reconstructed by advancing the lateral flap and employing the principle of "like with like" tissues and avoiding annexes with various skin textures

**Figure 6:**
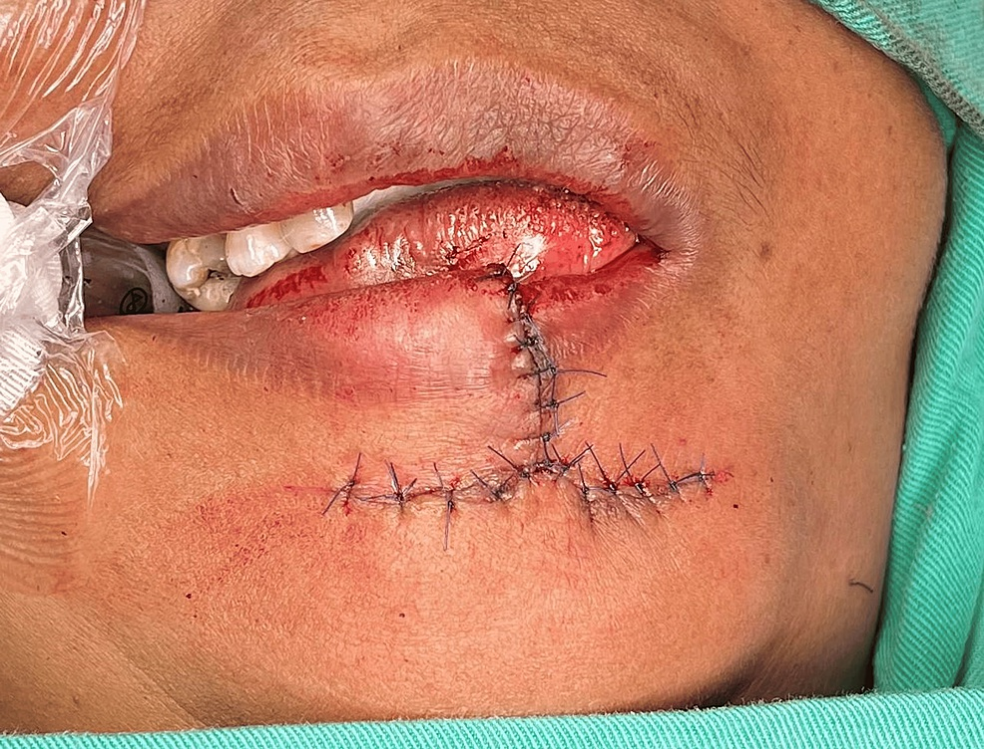
Full intraoperative closure

**Figure 7:**
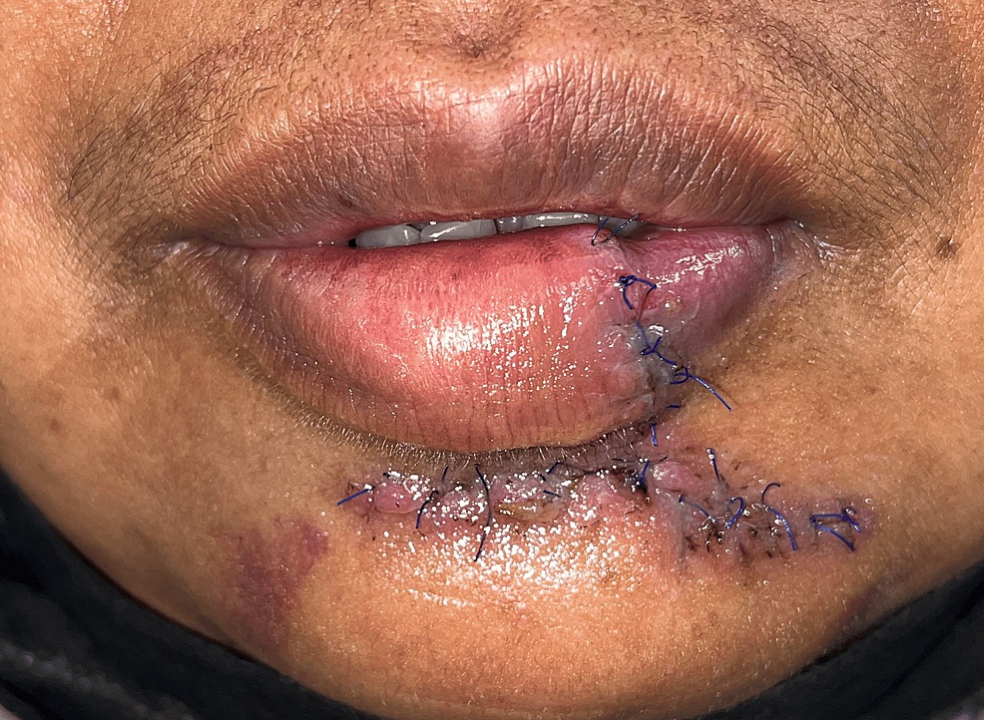
One week post-operation

**Figure 8:**
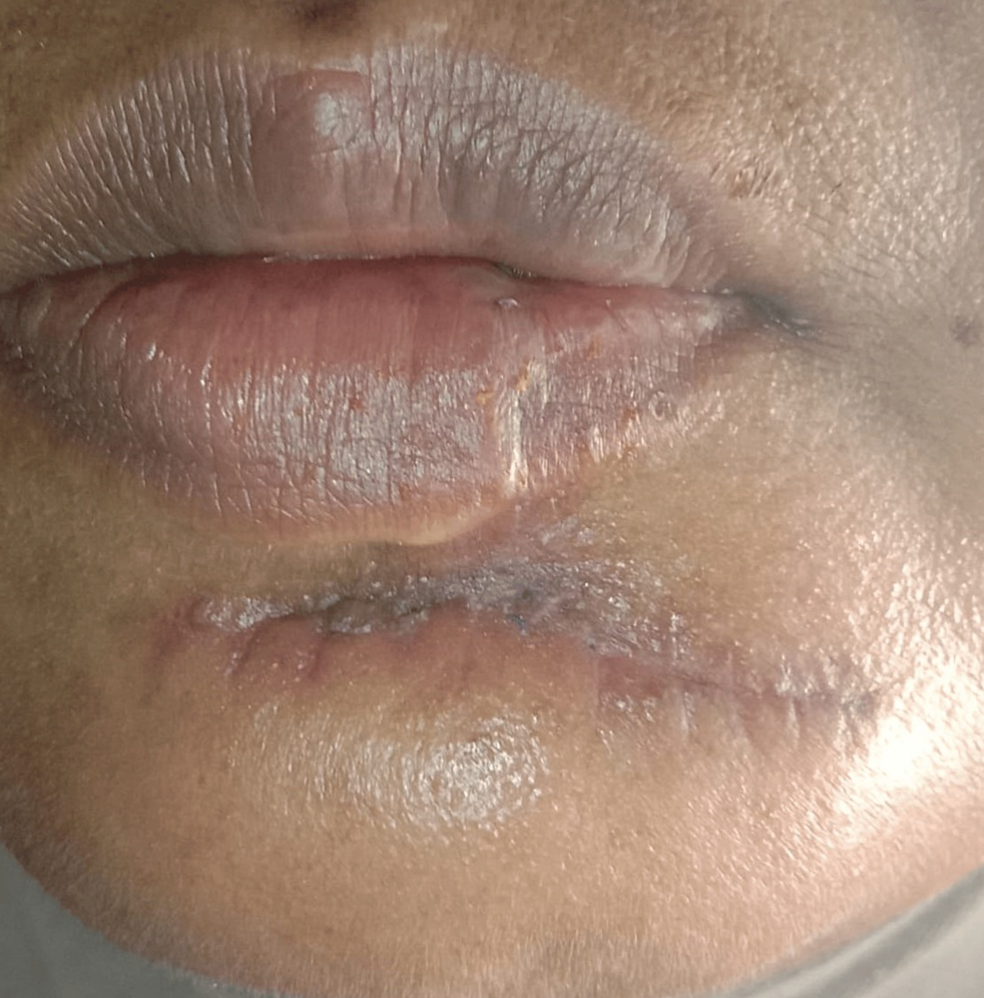
1 month post-operatively with minimal scarring and good post-operative healing.

**Figure 9:**
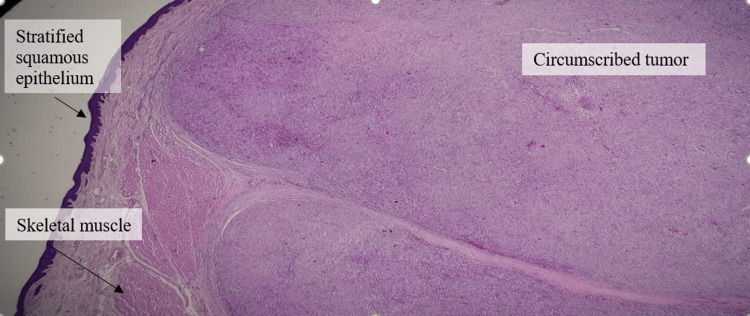
Circumscribed tumor beneath the lining epithelium within low-power magnification

**Figure 10:**
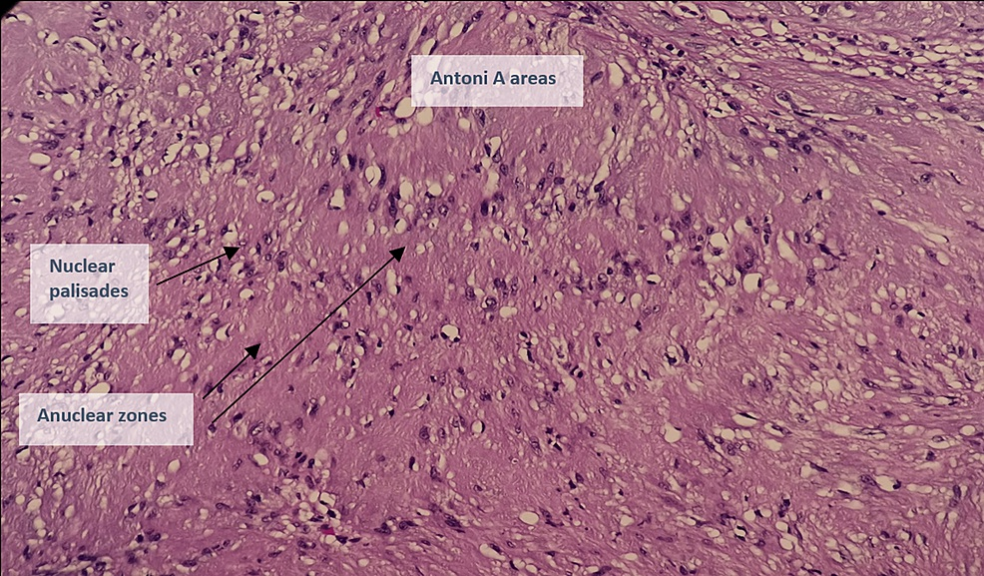
High-power magnification of the tumor showed a hypercellular (Antoni A) area composed of tumor cells with bland-looking, spindled to ovoid nuclei in a fascicular pattern. There are also Verocay bodies (nuclear palisades with anuclear zones).

**Figure 11:**
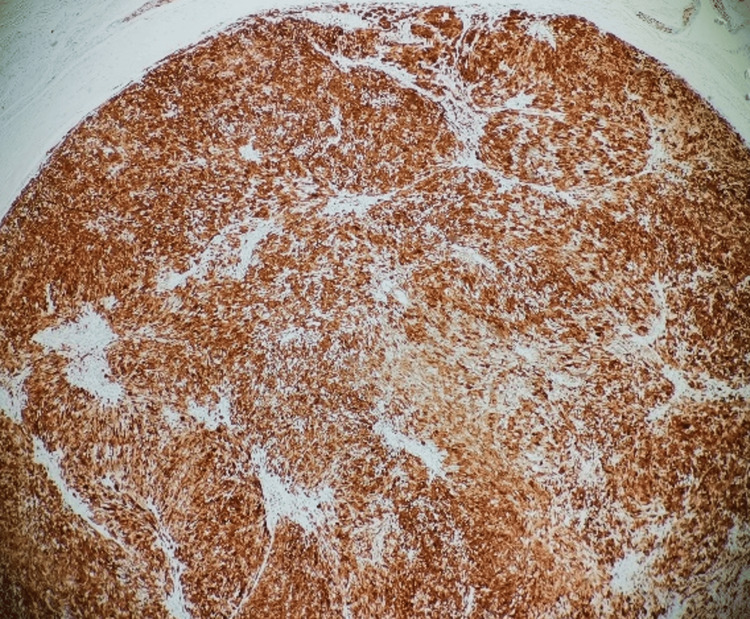
Low-power magnification showed that the tumor has a diffuse and strong positivity for S100

**Figure 12:**
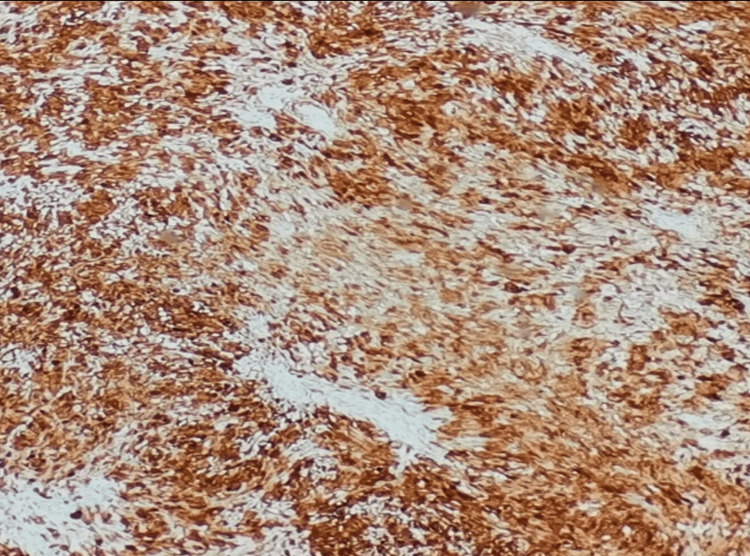
High-power magnification showed the tumor has a diffuse and strong positivity for S100

The swelling was firm in consistency and non-tender. There were no punctum, ulceration, or skin changes seen. The oral cavity and intraoral mucosa were unremarkable. There was no cervical lymphadenopathy. Fine-needle aspiration cytology of the mass was suggestive of a spindle cell lesion. Subsequently, she underwent an excision biopsy of the left lower lip and bilateral advancement flap closure, as in Figures 3-6. The intraoperative period was uneventful. Postoperatively, the wound healed with minimal scarring, and there was no recurrence on further follow-up (Figure 7 and Figure 8). The tissue sample was then sent for histopathological examination (Figures 9-12).

Histopathology revealed a circumscribed tumor composed of predominantly hypercellular (Antoni A) with a fascicular pattern of cells with bland-looking spindled to ovoid nuclei within a collagenous background. Verocay bodies are seen. Immunohistochemistry showed strong and diffuse positivity for S100 protein (Figures 9-12).

## Discussion

Most tumors on peripheral nerves are benign. They consist of neurofibroma and schwannoma (neurilemmoma) [1], both of which originate from Schwann cells [1]. These tumors were initially referred to as "neurinomas" when they were first described by Verocay in 1910. Due to the nerve sheath components connected to these tumors, the term "neurilemmoma" was proposed in 1935. The lesion's origin is still unknown; however, the perineurium's Schwann cell proliferation may be to blame.

In the fourth week of embryological development, the neural crest's ectomesenchymal cells gave rise to Schwann cells [2]. Schwann cells surround every axon in the peripheral nervous system. They support metabolic and structural processes. Schwann cells create a thin barrier around each extracranial nerve fiber to improve nerve conductivity and wrap bigger fibers in an insulating membrane to form the myelin sheath [3]. They create the neural sheath of the cranial, autonomic, and peripheral nerves [4].

The VIII cranial nerve (acoustic neuromas) is the most frequently impacted [5]. The head and neck regions are responsible for the development of 25% of all extracranial schwannomas; yet, only 1% of schwannomas originate from within the oral cavity, despite the fact that both the lips and the oral cavity are highly innervated anatomical areas [6]. The tongue has the highest incidence at the intraoral location, followed by the palate, buccal mucosa, lip, and gingiva [2]. In the clinical setting, schwannomas are typically asymptomatic and have a slow growth rate [7].

Histologically, they are easily distinguishable from other lesions. The tumor is composed of fibrocellular bundles forming a whorled pattern. There are two types of tissue arrangement: Antoni-A and Antoni-B. An alternation between Antoni A and B regions is common [7].

There are areas of dense and compact cellularity (Antoni A pattern) alternating with loose acellular areas (Antoni B pattern). Areas of the Antoni A pattern show palisaded nuclei called Verocay bodies. Nerve fibers are usually stretched over the capsule but not within the tumor as in Figures 9, 10*.*

Schwann cells characteristically express S-100 protein [1], as all neural origin tumors show positive for S-100 protein, as shown in Figures 11, 12. This stain can also differentiate benign nerve sheath tumors (S100 strong and diffuse) from malignant peripheral nerve sheath tumors (typically weak or negative S100) [8].

The distinction between neurofibroma and schwannoma is of the utmost importance. Physically, it may be challenging to differentiate between the two. However, there are histological and genetic differences. Schwannoma develops from Schwann cells, whereas neurofibroma arises in the fibroblasts found in the perineurium.

A detailed physical examination is warranted for any patient with schwannoma to rule out lesions in other parts of the body despite, in most cases, it being a unilocular lesion. However, a solitary neurofibroma may be the only presenting symptom of neurofibromatosis. Imaging methods like ultrasound, computed tomography (CT), and magnetic resonance imaging (MRI) are helpful for diagnostic objectives, including measuring tumor boundaries and lesion makeup and evaluating infiltration into surrounding structures [9,10].

Excision is the recommended modality of treatment. A functional surgery can be performed if the nerve can be successfully preserved and dissected away from the schwannoma during surgery to maintain the nerve function [9]. Another modality of treatment for schwannoma is stereotactic radiosurgery. This modality is a common treatment option for vestibular schwannomas [11].

Malignant transformation and recurrence of the disease is extremely rare. Malignant transformation for schwannoma is very rare, with the incidence of malignant nerve sheath tumors is 0.03 cases per 100,000/year [12,13].

This case report hopes to enlighten its readers on schwannomas as differentials in a painless swelling, especially in the head and neck region. A thorough examination must be carried out to arrive at a conclusive diagnosis. Proper surgical planning and postoperative monitoring are essential for good wound healing and complete recovery.

## Conclusions

The author of this case report hopes to enlighten its readers regarding schwannoma. It should be included as one of the differentials of a case of swelling within the head and neck region. Proper history and clinical and histopathological examination are key to establishing the diagnosis. Although it is rare, the location of such a swelling plays an essential role, as surgical excision is the best treatment modality. Even so, conservative monitoring is an option, especially for high-risk anesthesia patients, as malignancy is rare. Thus, the postoperative strategy focuses on recurrence recognition, healing, and cosmetic recovery.
